# Effects of acetyl-dl-leucine in patients with cerebellar ataxia: a case series

**DOI:** 10.1007/s00415-013-7016-x

**Published:** 2013-07-09

**Authors:** Michael Strupp, Julian Teufel, Maximilian Habs, Regina Feuerecker, Carolin Muth, Bart P. van de Warrenburg, Thomas Klopstock, Katharina Feil

**Affiliations:** 1 Department of Neurology, University Hospital Munich and German Center for Vertigo and Balance Disorders, Campus Grosshadern, Marchioninistrasse 15, 81377 Munich, Germany; 2 Department of Neurology, Donders Institute for Brain, Cognition, and Behaviour, Radboud University Nijmegen Medical Centre, Nijmegen, Netherlands; 3 Friedrich-Baur-Institute, German Network for Mitochondrial Disorders (mitoNET) and German Center for Neurodegenerative Diseases–DZNE, Munich, Germany

**Keywords:** Ataxia, Cerebellum, Cerebellar ataxia, Acetyl-dl-leucine, Pharmacotherapy

## Abstract

No existing medication has yet been shown to convincingly improve cerebellar ataxia. Therefore, the identification of new drugs for its symptomatic treatment is desirable. The objective of this case series was to evaluate the efficacy of treatment of cerebellar ataxia with the amino acid acetyl-dl-leucine (Tanganil). Thirteen patients (eight males, median age 51 years) with degenerative cerebellar ataxia of different etiologies (SCA1/2, ADCA, AOA, SAOA) were treated with acetyl-dl-leucine (5 g/day) without titration for 1 week. Motor function was evaluated by changes in the Scale for the Rating and Assessment of Ataxia (SARA) and in the Spinocerebellar Ataxia Functional Index (SCAFI) during treatment compared to a baseline examination. Quality of life (EuroQol-5D-3L) and side effects were also assessed. Mean total SARA decreased remarkably (*p* = 0.002) from a baseline of 16.1 ± 7.1 to 12.8 ± 6.8 (mean ± SD) on medication. There were also significant improvements in sub-scores for gait (*p* = 0.022), speech (*p* = 0.007), finger-chase (*p* = 0.042), nose-finger-test (*p* = 0.035), rapid-alternating-movements (*p* = 0.002) and heel-to-shin (*p* = 0.018). Furthermore, patients showed better performance in the SCAFI consisting of the 8-m-walking-time (8 MW, *p* = 0.003), 9-Hole-Peg-Test of the dominant hand (9HPTD, *p* = 0.011) and the PATA rate (*p* = 0.005). Quality of life increased during treatment (*p* = 0.003). No side effects were reported. In conclusion, acetyl-dl-leucine significantly improved ataxic symptoms without side effects and therefore showed a good risk–benefit profile. These findings need to be confirmed in placebo-controlled trials.

## Introduction

Cerebellar ataxia is a frequent and often-disabling syndrome that is mainly characterized by limb and gait ataxia as well as speech and ocular function impairment. In the majority of cases, it is caused by progressive heredodegenerative disorders [1]. So far, treatment of cerebellar ataxia has remained a difficult task as no medication has yet been proven really effective. There were attempts in the past to treat cerebellar signs, for example downbeat nystagmus (DBN) [2], episodic ataxia type 2 (EA2) [3], or cerebellar gait ataxia [4] with aminopyridines. Physiotherapy is recommended and applied in clinical practice but has only moderate effects [5–7]. Therefore, new drugs are needed, as cerebellar ataxia often leads to a significant disability of the affected patients.

Since 1957, acetyl-dl-leucine (Tanganil; Pierre Fabre, Castres, France), an acetylated derivative of a natural amino acid, has been widely used—mainly in France—for the symptomatic treatment of acute vertigo and dizziness and to improve central vestibular compensation [8]. Clinical experience has shown that it is a well-tolerated and safe drug without serious adverse effects [9–11].

Electrophysiological measurements have demonstrated that acetyl-dl-leucine modulates the activity of central vestibular neurons by normalizing the membrane potential of depolarized or hyperpolarized neurons [12], a mechanism that may lead to the observed improvement of central compensation. Furthermore, clinical studies with branched-chain amino acids demonstrated a symptomatic improvement of cerebellar symptoms [13]. As acetyl-dl-leucine is a branched-chain amino acid and in view of the phylogenetic and electrophysiological similarities and close interactions between vestibular and cerebellar neurons [14], we hypothesized that this agent might have a positive effect on ataxic symptoms in cerebellar disorders.

## Design/methods

Thirteen patients (eight males; median age 51 years, range 13–68; median symptom duration 3 years, range 2–25 years; for further details see Table 1) were treated with acetyl-dl-leucine 5 g/day without titration (500 mg tablets of Tanganil) for 7 days. All patients gave their informed consent for the compassionate use of acetyl-dl-leucine. The measurements were based on the Scale for the Rating and Assessment of Ataxia (SARA) [15, 16], an eight-item clinical rating scale (gait, stance, sitting, speech, coordination; range 0–40), and the Spinocerebellar Ataxia Functional Index (SCAFI) [17, 18], which consists of the 8-m-walking-time (8MW), 9-Hole-Peg-Test (9HPT) for appendicular function and the number of “PATA” repetitions over 10 s (PATA). These tests were performed at baseline and during treatment (on day 9 ± 3 days).

**Table 1:** Patient characteristics, categorized by patient number, gender, age, etiology, age at onset, duration of disease, MRI, and neuro-ophthalmological findings

Patient no.	Sex	Age	Type	Age at onset	Duration (in years)	Brain MRI findings	Neurological findings
1	Male	60	SAOA	58	2	Normal	1, 5 (unilateral), 6, 7, horizontal hypermetric saccades, square wave jerks
2	Male	51	SAOA	48	3	Normal	1, 2, 3, 6, 7 hypometric saccades
3	Male	54	SAOA	51	3	Normal	1, 2, 7, 8
4	Female	63	SAOA	38	25	Atrophy of vermis	1, 2, 3, 4, 6, 7
5	Male	23	SCA2	20	3	Atrophy of cerebellum	1, 2, 7, slow saccades
6^a^	Female	57	ADCA	44	9	Atrophy of the vermis	1, 3
7^a^	Female	68	ADCA	54	14	Atrophy of the vermis	1, 2, 3, 6, 7, 8, hypometric saccades, SVV deviation
8	Female	47	SCA1	44	3	Atrophy of the cerebellum	1, 2, 5 (bilateral), 6, 7, hypermetric saccades
9	Female	56	SAOA	54	2	Atrophy of the vermis and anterior lobe	1, 6
10^b^	Male	25	AOA	12	13	Atrophy of cerebellum	1, 2, 7 PNP, muscle atrophy, dysarthrophonia, ocular apraxia
11^b^	Male	23	AOA	11	12	Atrophy of cerebellum	1, 2, 7 PNP, muscle atrophy, dysarthrophonia, ocular apraxia
12^b^	Male	19	AOA	10	9	Atrophy of cerebellum	1, 7 PNP, muscle atrophy, dysarthrophonia, ocular apraxia
13^b^	Male	13	AOA	11	2	Atrophy of cerebellum	7 Dysarthrophonia

*AOA* Ataxia with oculomotor apraxia (AOA1 and AOA2 genetically excluded), *SAOA* sporadic adult-onset ataxia of unknown etiology, *ADCA* autosomal dominant cerebellar ataxia, *SVV* subjective visual vertical, *PNP* polyneuropathy, *1* saccadic smooth pursuit, *2* gaze-evoked nystagmus, *3* head-shaking nystagmus, *4* rebound nystagmus, *5* pathological head-thrust test (uni- or bilateral), *6* impaired visual fixation suppression of the VOR, *7* pathological optokinetic reflex, *8* downbeat nystagmus

^a^Relatives of family one (cousins)

^b^Relatives of family two (siblings)

Quality of life during this symptomatic treatment was evaluated by using the Euro-Qol-5D-3L questionnaire (EQ-5D-3L) [19]. Patients were asked about their subjective improvement on medication. The known side effects such as skin reactions (rash, urticaria, itching) were also systemically evaluated. Statistical analysis was performed using SPSS (IBM, Armonk, NY). Figures were designed with GraphPad Prism (v5, GraphPad Software Inc., La Jolla, CA, USA). Differences were considered significant if *p* < 0.05. As data were not normally distributed, non-parametric testing was performed (Wilcoxon signed-rank test) with a Bonferroni correction.

## Results

**Fig. 1:**
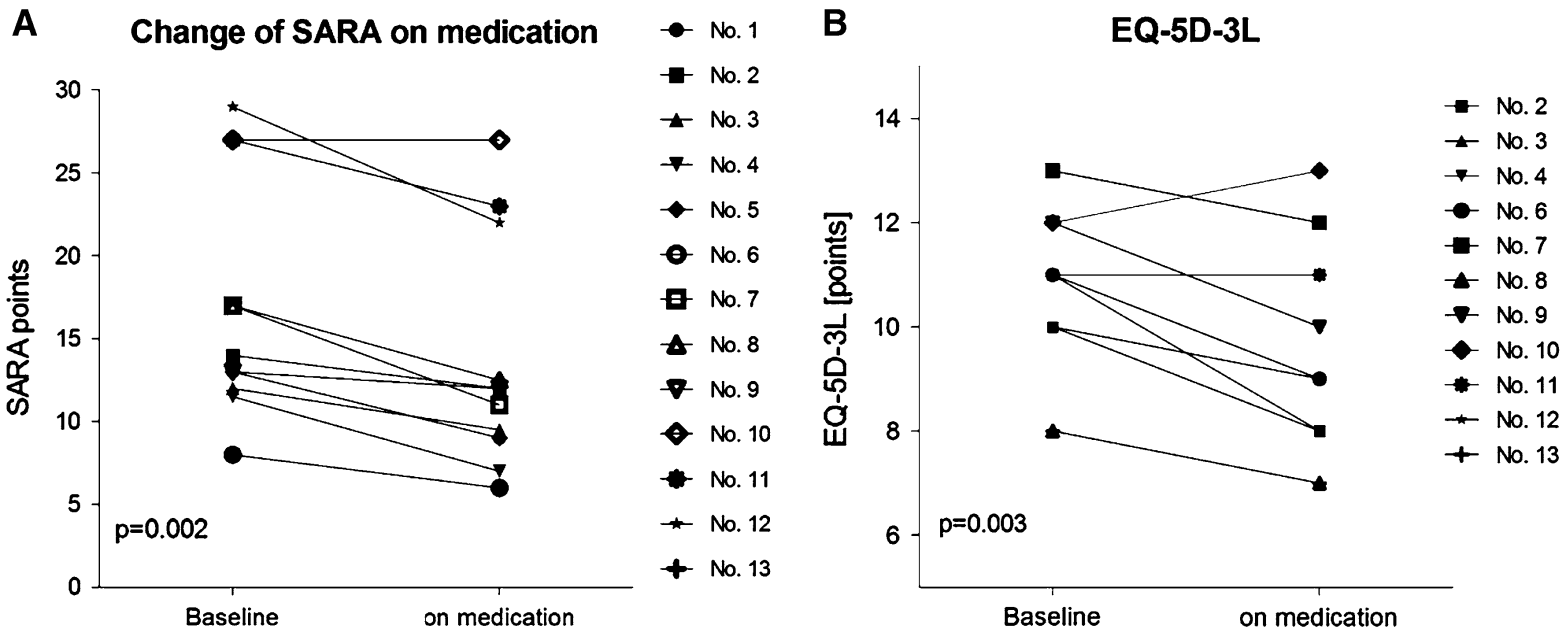
Individual changes on **a** Scale for the Rating and Assessment of Ataxia (SARA) and **b** Quality of Life Questionnaire Euro-Qol-5D-3L (EQ-5D-3L) on medication with acetyl-dl-leucine (5 g/day) for 9 ± 3 days

**Fig. 2:**
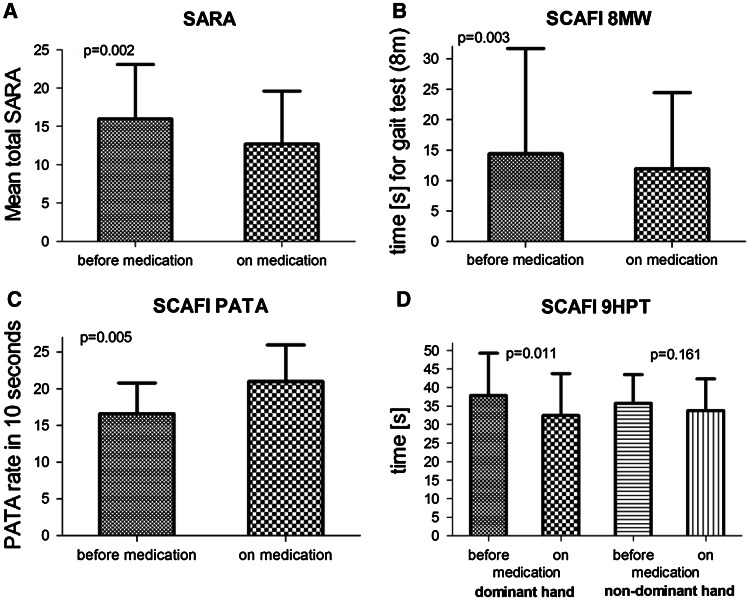
Value changes on **a** Scale for the Assessment and Rating of Ataxia (SARA) and Spinocerebellar Ataxia Functional Index (SCAFI) sub-score items in terms of **b** 8 m walk (8 MW), **c** PATA word count in 10 s and **d** 9-hole-peg-test (9HPT) of the dominant and non-dominant hand before and during the therapy with acetyl-dl-leucine (5 g/day) (mean ± SD)

The clinical characteristics of the patients are given in Table 1. Thirteen patients with cerebellar disorders of different etiologies were observed (since patient no. 1 did not perform the SCAFI, he was excluded from the statistical analysis of this test). Mean duration of cerebellar symptoms was 8.5 ± 6.8 years. Mean total SARA decreased from a baseline of 16.1 ± 7.1 to 12.8 ± 6.8 (*p* = 0.002, Wilcoxon signed-rank test). During treatment the SARA subsection scores were notably lower for gait (*p* = 0.022), speech disturbance (*p* = 0.007), finger-chase (*p* = 0.042), nose-finger-test (*p* = 0.035), rapid-alternating-hand-movements (*p* = 0.002) and heel–shin-slide (*p* = 0.018) (Table 2; Figs. 1, 2). The SCAFI items also improved, represented by the 8 MW (*p* = 0.003), the 9HPT of the dominant hand (*p* = 0.011) and the PATA rate (*p* = 0.005). Moreover, the EQ-5D-3L changed from a baseline of 10.7 ± 1.6 to 9.5 ± 2.0 (*p* = 0.003), indicating that quality of life was higher during treatment. The visual analogue scale (VAS)—as part of the EQ-5D-3L—rose from 0.57 ± 0.21 to 0.63 ± 0.19 (*p* = 0.241). Eleven out of 13 patients reported a subjective improvement on therapy (nos. 1, 3, 4, 6, 7, 8, 9, 10, 11, 12, 13). The patients did not report any side effects.

**Table 2:** Clinical assessment of patients by SARA and SCAFI at baseline and on medication

Patient no.	Baseline					On medication				
	SARA	8 MW	PATA	9HPT-D	9HPT-N	SARA	8 MW	PATA	9HPT-D	9HPT-N
1	13.00	–^c^	–^c^	–^c^	–^c^	9.00	–^c^	–^c^	–^c^	–^c^
2	14.00	5.10	20.00	32.00	38.00	12.00	4.80	26.00	21.00	41.00
3	12.00	4.90	14.00	41.20	38.20	9.50	4.70	22.00	37.80	33.80
4	11.50	8.60	15.00	28.80	30.40	7.00	8.10	24.00	26.80	27.10
5	13.00	5.20	23.00	41.40	40.70	9.00	4.40	23.00	30.40	29.90
6^a^	8.00	6.60	16.00	25.90	25.40	6.00	6.00	24.00	24.60	25.40
7^a^	17.00	10.10	19.00	37.60	40.70	11.00	8.00	22.00	29.70	38.70
8	17.00	14.60	24.00	62.00	49.80	12.50	12.70	25.00	59.10	51.70
9	13.00	6.70	14.00	45.00	30.70	12.00	6.50	26.00	35.70	27.80
10^b^	27.00	–^d^	11.00	–^d^	–^d^	27.00	–^d^	11.00	–^d^	–^d^
11^b^	27.00	62.20	17.00	–^d^	–^d^	23.00	44.00	18.00	–^d^	–^d^
12^b^	29.00	28.60	11.00	–^d^	–^d^	22.00	27.30	13.00	–^d^	–^d^

*SARA* Scale for the Assessment and Rating of Ataxia, *SCAFI* Spinocerebellar Ataxia Functional Index, *8* *MW* 8-m-walking-time, *9HPTD* 9-Hole Peg Test of the dominant hand, *9HPTN* 9-Hole Peg Test of the non-dominant hand

^a^Relatives of family one (cousins)

^b^Relatives of family two (siblings)

^c^Not performed for other reasons

^d^Not able to perform due to disabling reasons

## Discussion

The 13 patients in this case series had ataxia as the leading symptom of cerebellar disorders of different etiologies. Patients on medication with acetyl-dl-leucine showed a significant improvement of both the ataxic symptoms—measured by SARA and SCAFI—as well as quality of life. The daily administration of 5 g/day used in this case series is in the upper range of the drug’s recommended dosage, but lower than in studies on its use for acute vertigo [8 g/day; for reference see (https://www.clinicaltrialsregister.eu/ctr-search/trial/2010-023099-13/DE)]. As expected from its safety profile, acetyl-dl-leucine was well tolerated; patients did not report any side effects.

Currently there is no proven symptomatic pharmacotherapy for cerebellar ataxias. Drugs such as varenicline [20], riluzole [21] or erythropoietin in Friedreich’s ataxia [22] were tested in the past. In a placebo-controlled trial with varenicline SCA3 patients showed an improvement of −1.97 SARA points compared to −0.86 on placebo [20]. As mentioned above, aminopyridines are effective in a subset of symptoms (DBN [2] or EA2 [3]). Because there are no effective anti-ataxia drugs, current standard treatment recommendations mainly include physiotherapy [5, 7]. The improvement of function after 4 weeks of intensive physiotherapy was −5.2 SARA points [5]; patients regained functional performance equivalent to that after two or more years of disease progression. The natural disease progression of degenerative cerebellar ataxias is 0.6–2.5 SARA points per year [5]. Compared to this, patients on treatment with acetyl-dl-leucine had an improvement of −3.3 SARA points after treatment for 9 ± 3 days. It has to be mentioned that the patient group in this case series was inhomogeneous—so it is probable that the positive effects might be even be better in a more homogenous group of patients.

Although acetyl-dl-leucine has been in use for more than 50 years, its exact pharmacological and electrophysiological modes of action have not been fully elucidated. An animal model of acute unilateral labyrinthectomy in guinea pigs showed that acetyl-dl-leucine restored the membrane potential of both hyperpolarized and depolarized vestibular neurons [8, 12]. This effect is assumed to be mediated by its direct interactions with membrane phospholipids [11] such as phosphatidylinositol 4,5-bisphosphate, which influences ion channel activity [23]. On the basis of these findings, acetyl-dl-leucine may also stabilize the membrane potential of cerebellar neurons. The input from cerebellar Purkinje cells and mossy/climbing fiber collaterals controls the action potential of cerebellar and vestibular nuclei [24], which in turn project to the brainstem, thalamus and spinal cord [14]. Thus, acetyl-dl-leucine may act through afferent and efferent projections on upstream and downstream structures, thereby influencing motor control. It may also influence adaptive mechanisms throughout the vestibular–cerebellar network and be involved in the balancing of information between the cerebellum and effector muscles. However, the modes of action of acetyl-dl-leucine in cerebellar disorders are still largely hypothetical and require further research. Our clinical findings on its efficacy in cerebellar ataxia must be carefully evaluated in electrophysiological studies and animal models of cerebellar ataxia as well as placebo-controlled and PET imaging studies in humans.

Clearly this case series has several limitations. First, this is not a randomized, placebo-controlled trial. Second, the change in the symptoms after termination of the treatment was not systematically evaluated. Third, we cannot rule out either a placebo effect or a training effect on components of the ataxia assessment (e.g., 9HPT), but neither effect can explain the significant and clinically convincing effects documented by the standard ataxia scores SARA and SCAFI. In conclusion, its improvement of cerebellar ataxia and the absence of adverse effects indicate that acetyl-dl-leucine is a worthy candidate for further clinical trials and also basic research.
